# Do anti-VEGFs used in the ophthalmic clinic cause Müller glial cell stress?

**DOI:** 10.1016/j.clinsp.2022.100161

**Published:** 2023-01-24

**Authors:** Rafael André da Silva, Luiz Philipe de Souza Ferreira, Vinicius Moraes de Paiva Roda, José Maria Soares Junior, Manuel de Jesus Simões, Caio Vinicius Saito Regatieri

**Affiliations:** a Biosciences Graduate Program, Institute of Biosciences, Instituto de Biociências, Letras e Ciências Exatas, Universidade Estadual Paulista (IBILCE/UNESP), São José do Rio Preto, SP, Brazil; b Structural and Functional Biology Graduate Program, Escola Paulista de Medicina, Universidade Federal de São Paulo (EPM/UNIFESP), São Paulo, SP, Brazil; c Life Systems Biology Graduate Program, Instituto de Ciências Biomédicas, Universidade de São Paulo (ICB/USP), São Paulo, SP, Brazil; d Department of Obstetrics and Gynecology, Faculdade de Medicina, Universidade de São Paulo (FMUSP), São Paulo, SP, Brazil; e Department of Ophthalmology, Escola Paulista de Medicina, Universidade Federal de São Paulo (EPM/UNIFESP), São Paulo, SP, Brazil

Age-related Macular Degeneration (AMD) and Diabetic Retinopathy (DR) are eye diseases that can lead to vision loss. AMD mainly affects the elderly and DR individuals of different ages.^1^^,^^2^ Müller Glial Cells (MGCs) play a crucial role in the pathogenesis of these diseases, modulating inflammation and angiogenesis.^3^^,^^4^ Activated MGCs in gliosis overexpress Glial Fibrillary Acid Protein (GFAP) and actively produce Vascular Endothelial Growth Factor (VEGF), leading to abnormal retinal angiogenesis and microinflammation.^2^^,^^5^ To manipulate the main signaling pathway involved in neovascular AMD and DR, anti-VEGF drugs are used intravitreally in the ophthalmic clinic, including ranibizumab, bevacizumab, aflibercept and brolucizumab.^1^ However, little is known about the impact of anti-VEGF medications on Müller cells.

In vitro, primary MGC cells, retinal cells in organotypic culture, as well as human Müller cells line (MIO-M1) treated with aflibercept, ranibizumab, or bevacizumab show time-dependent increased GFAP expression.^6^, ^7^, ^8^, ^9^ Aflibercept and Ranibizumab regulate GFAP positively via pERK1/2.^6^ In rabbits, ziv-aflibercept, although it does not change the electroretinogram, increases the expression of GFAP, suggesting retinal stress caused by the drug.^10^

MIO-M1 cells, treated during 24 h with anti-VEGF drugs, show a reduction of these cells’ metabolism. Additionally, there is an increase of reactive oxygen species and expression of the pro-inflammatory interleukin IL-β which are apoptosis markers.^11^ Bevacizumab positively regulates the apoptosis of Müller cells via caspase-3.^7^ Conversely in another study, aflibercept and ranibizumab in 24 h do not affect cell survival. Furthermore, mitochondrial and cytoplasmic stress were observed through HSP60 and HSP90 in MIO-M1 cells.^12^

Anti-VEGF drugs are efficient to treat AMD and DR,^1^^,^^13^ therefore, so far, through the data available in the literature, we must consider that Müller cells can undergo cellular stress, evidenced by the main gliosis marker, GFAP. In addition, anti-VEGF drugs can disrupt the metabolism of these cells. It is important at this time that we carry out translational studies in humans to investigate the points highlighted here. Cohort studies, considering the long treatment inpatients, show retinal atrophy and fibrosis,^14^ which may be related to gliosis and resistance to anti-VEGFs in some patients with AMD and RD.^1^

We can find different results of MGCs’ gliosis and survival under the influence of anti-VEGF drugs; this may be related to the particular molecular mechanism of each drug. Among the anti-VEGF drugs used in the ophthalmic clinic for AMD and PDR, brolucizumab was not investigated in MGCs. Although we have evidence that anti-VEGF drugs can lead to gliosis of MGCs, more studies are needed to understand the mechanisms around this stress. It is essential to understand these mechanisms for a possible improvement of existing drugs and facilitate new therapeutic interventions with fewer side effects.

## Conflicts of interest

The authors declare no conflict of interest.
